# Supplementary material for: Sub-harmonic entrainment of cortical gamma oscillations to deep brain stimulation in Parkinson's disease: Model based predictions and validation in three human subjects
Source: Brain Stimul. 2023 Sep-Oct;16(5):1412–24. doi: 10.1016/j.brs.2023.08.026 (PMC10635843; doi:10.1016/j.brs.2023.08.026)
Supplement: Supplementary Materials — Further details of the optimisation process, as well as the off-stimulation data and features from the other two patients, the fitting robustness, entrainment predictions for different stimulation patterns, and phase-locking of entrained data signals, are presented here. [file mmc1.pdf]

# Supplementary material

# A Optimisation Details

## A.1 Correlation and Redundancy Between Features

To see consistent population dynamics and entrainment predictions it is required to fit the Wilson-Cowan model parameters to all three features mentioned in the *Fitting Process* section.

We demonstrated this by also fitting to each of the three features individually (approximately 1000 local optimisations per feature). The costs of these optimisations, along with the costs from the optimisation of all three features, are plotted in Fig S.1. In each panel, the trade-off between pairs of features is represented by the Pareto front. As expected, optimising only one feature results in low costs for this feature and significant variability for the other two costs. The results of the optimisation to all three features may not have costs as low as the fits to an individual feature, but they are consistently in the bottom most left corner of the plot and the best trade off between optimising each feature. Moreover, the results of the fits to individual features had greater variability in the entrainment field predictions than the fit to all three features.

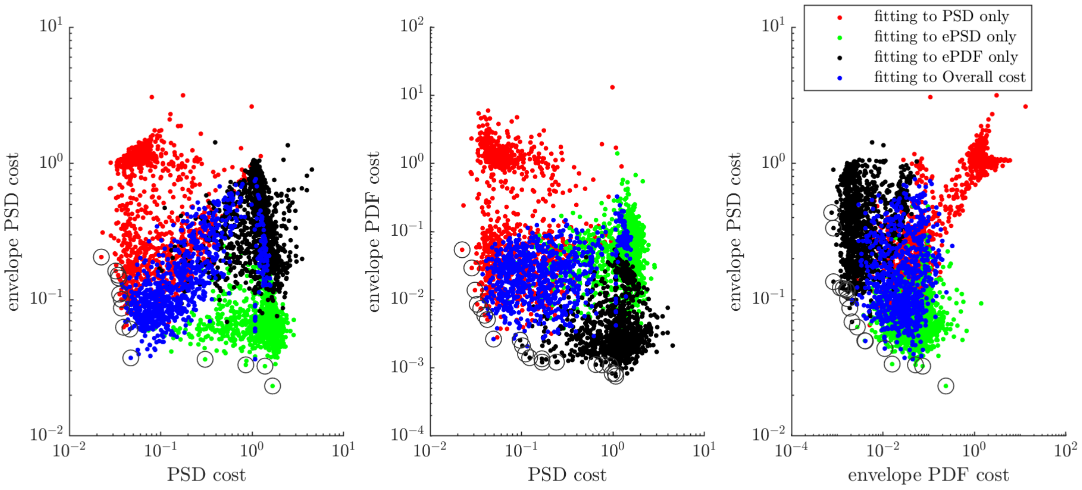


**Figure S.1: Feature costs when fitting to a single feature only, compared to fitting to all features.** Results of fitting to just the PSD (red), envelope PSD (green), envelope PDF (black), and all three features (blue). Panel A shows the comparison of the PSD cost to envelope PSD cost, panel B PSD to envelope PDF cost, and panel C envelope PDF to envelope PSD cost. In all panels, the Pareto front is highlighted by grey rings.

It does not appear that any of these features are redundant to model off-stimulation local field potentials as shown by the low correlation (there is little overlap of costs for the four optimisations in Fig S.1) and the Pareto front in the three panels of Fig S.1. Hence, to capture the full time-domain-based dynamics it is necessary to fit to all three features.

## A.2 Evaluation of Cost

For each parameter set, the model is forward simulated using the Euler-Maruyama scheme for a four second time period, with an additional 0.1 second settling period, and a time step of $10^{-4}$ seconds. Following the model simulation, the transient of the settling period is removed and the population activity is z-scored. The three features are then computed in the exact same manner as the data features. The overall cost against the data is calculated by

$$\text{cost} = \frac{1}{3}\sum_{n=1}^{3}\left(\frac{\sum_{i=1}^{L_n}(X_{n,i}^{\text{data}} - X_{n,i}^{\text{model}})^2}{\sum_{i=1}^{L_n}(X_{n,i}^{\text{data}} - \overline{X_n^{\text{data}}})^2}\right),$$

where $n = [1, 2, 3]$ for the three features and $L_n$ is the length of each feature, $X_n$. The optimiser then looks to minimise the cost, which maximises the coefficient of determination, $R^2 = 1 - \text{cost}$.

### A.3 Robustness to Noise

Stochasticity introduces the possibility during optimisation that for a given simulation, the randomly generated noise vector may be particularly favourable to minimise the feature costs. However, this parameter set would not represent a local minimum on average. The four second time period of model forward simulation is chosen as a trade off between minimal run time and sufficient individual simulations to minimise the effect of variability due to stochasticity on the parameter set $R^2$. Following the evaluation of the best model parameter fits, each of the top 20 model parameter sets are simulated three additional times and the features calculated with a different random noise vector. This ensures that the optimal fit was not the result of a single, favourable noise vector. In RCS10 for example, the six top parameter sets featured in Fig E.1 all maintained an $R^2$ value above 0.92 across the three additional simulations.

### A.4 Top-Ranked Wilson-Cowan Model Parameter Set

Parameters of the top-ranked Wilson-Cowan model fit are given in Table A. The top-ranked parameter set from RCS18 had a smaller, but wider off-stimulation gamma peak making the final parameter ranking more prone to variation from noise (see Fig S.2B1). This introduce more variability in the entrainment predictions, but the 1:2 tongue was still consistently predicted to be left leaning in the top 20 parameter sets.

**Table A: Parameter set from the best ranked fits.** The given parameter sets had an average $R^2$ value of 0.961, PSD cost of 0.038, envelope PSD cost of 0.072 and PDF cost of 0.008 for RCS02, $R^2$ value of 0.944, PSD cost of 0.052, envelope PSD cost of 0.070 and PDF cost of 0.046 for RCS10, $R^2$ value of 0.868, PSD cost of 0.156, envelope PSD cost of 0.221 and PDF cost of 0.019 for RCS18, across the 50 simulations lasting 100 seconds each.

| Parameter | $\omega_{EE}$ | $\omega_{EI}$ | $\omega_{IE}$ | $\tau_E$ | $\tau_I$ | $\eta_E$ | $\eta_I$ | $\beta$ | $\zeta$ |
|---|---|---|---|---|---|---|---|---|---|
| RCS02 | 4.795 | 7.224 | 4.913 | $6.424 \cdot 10^{-3}$ | $4.853 \cdot 10^{-3}$ | 1.977 | -1.020 | 2.216 | 0.087 |
| RCS10 | 1.130 | 1.722 | 3.311 | $9.322 \cdot 10^{-3}$ | $7.329 \cdot 10^{-3}$ | 3.065 | 0.709 | 9.273 | 0.048 |
| RCS18 | 2.590 | 7.679 | 7.140 | $8.595 \cdot 10^{-3}$ | $1.131 \cdot 10^{-2}$ | 4.007 | -0.436 | 3.329 | 0.038 |

## B Clinical Symptoms of all Patients

The clinical UPDRS scores for each patient are given in Table B. The clinical effects of FTG entrainment were not explored here.

**Table B: Patient clinical symptom summary.** All assessments were done 90 days pre-operation. A few supplemental sub-scores are included (*MDS-UPDRS Part IV,** sum of all MDS-UPDRS-III tremor scores for each side, off medication).

| Patient | UPDRS III OFF-med | UPDRS III ON-med | % change UPDRS when ON | time with dyskineasia* | Functional impact of dykinesia* | OFF-med tremor score** |
|---|---|---|---|---|---|---|
| RCS02 | 49 | 5 | 90% | 4 | 0 | L: 2; R: 0 |
| RCS10 | 89 | 42 | 53% | 0 | 0 | L: 9; R: 11 |
| RCS18 | 30 | 7 | 76% | 2 | 1 | L: 1; R: 1 |

## C Off-Stimulation Data and Model Features in RCS18 and RCS02

Off-stimulation data features (PSD, envelope PSD and envelope PDF) are shown for RCS02 and RCS18 in Fig S.2. The features of the top ranked models fits are compared to data features for RCS02 and RCS18 in Fig S.3.

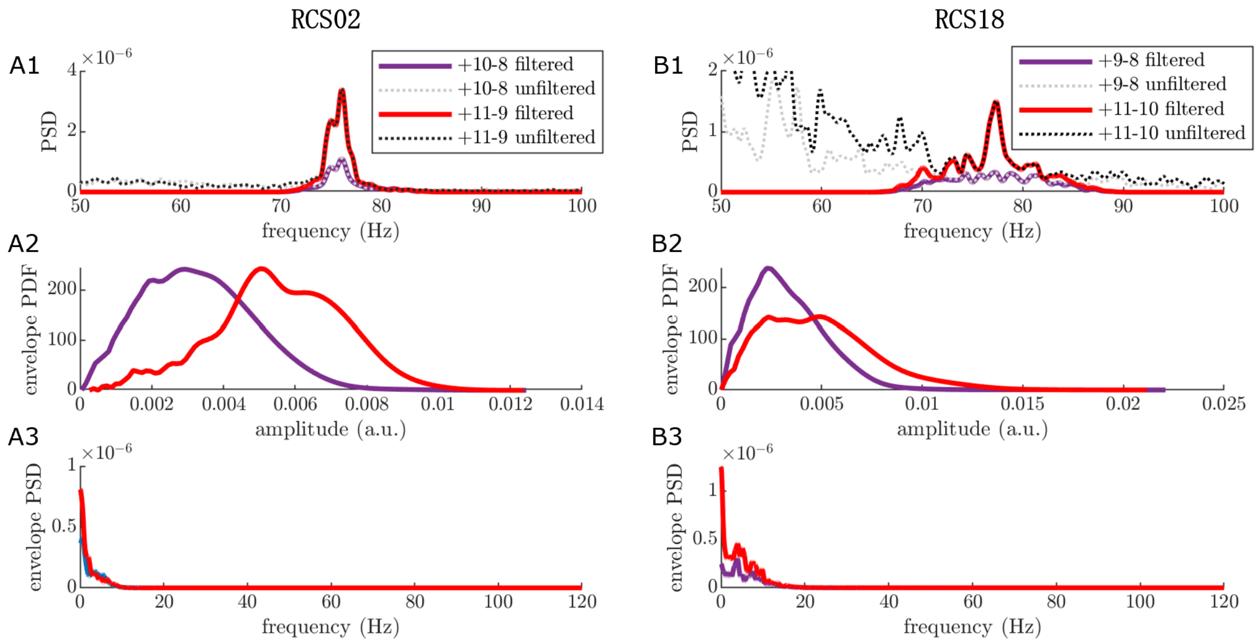


**Figure S.2: Features of prestimulation human cortical recordings to fit Wilson-Cowan model parameters in RCS02 and RCS18.** Off-stimulation features are obtained as described in the main text. The three data features are PSD (1), envelope PDF (2) and envelope PSD (3), for the selected epoch. Panels A1-3 are calculated from RCS02 cortical data filtered between 72 and 78Hz, as the gamma peak occurred at 75Hz. The fitting process for RCS02 was performed with the features from the 11-9 contact pair as the objective. Panels B1-3 are calculated from RCS18 cortical data filtered between 75 and 81Hz, as the gamma peak occurred at 78Hz. The fitting process for RCS18 was performed with the features from the 11-10 contact pair as the objective. The solid red and purple lines display the band-pass filtered cortical signals. The black and grey dotted lines in the PSD plot show the unfiltered cortical signals.

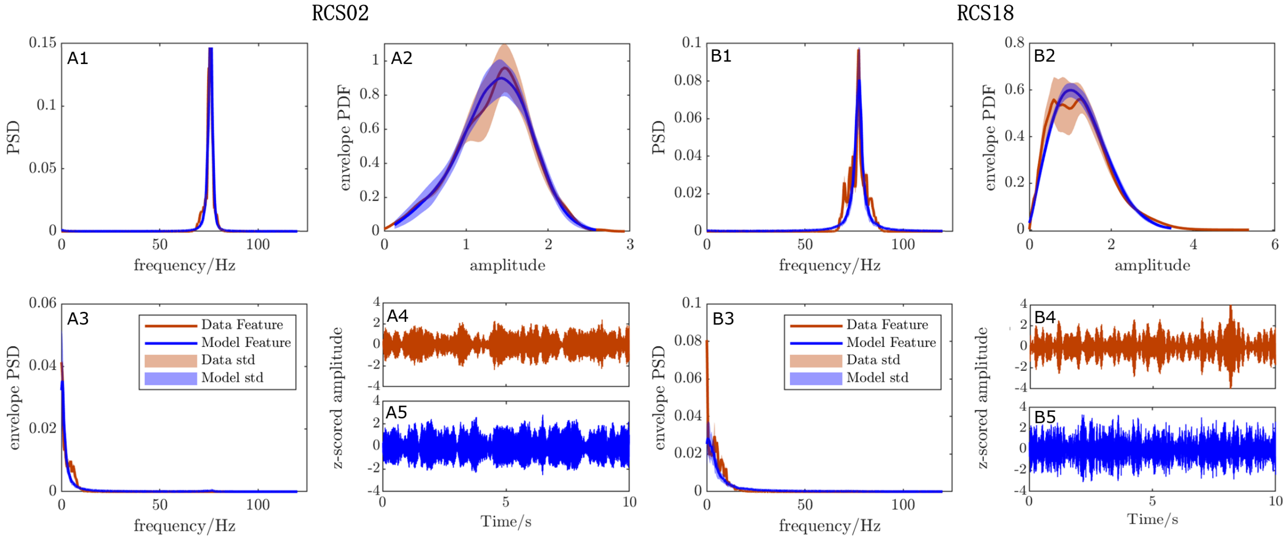


**Figure S.3: Comparison of the cortical FTG features for z-scored data and the features from the z-scored model output of the best ranked model parameter set for RCS02 and RCS18.** Panels A1-5 are the features for RCS02 and Panels B1-5 are the features for RCS18. $R^2 = 0.961$ for RCS02 and $R^2 = 0.868$ for RCS18 on average across 50 simulations lasting 100 seconds each. The features are PSD (1), envelope PDF (2), and envelope PSD (3). (4 and 5) Comparison of the band-passed, z-scored, off-stimulation time series from patient data (4) and model output (5) show that the model is capable of replicating similar time-domain dynamics. In all panels, the data is in red, and the model in blue.

# D Sine Circle Map

The sine circle map is the simplest model that describes the influence of periodic stimulation on an oscillator and can provide a first-level description of gamma entrainment during 130Hz stimulation. The model stroboscopically observes the phase, $\theta$, of a single oscillator of natural frequency $f_0$, periodically stimulated at frequency $f_s$ and stimulation intensity, $\mathrm{A}_s$. The map between the oscillator phase right after stimulation pulse $i$ and its phase right after stimulation pulse $i+1$ is given by

$$\theta_{i+1} = \theta_i + 2\pi(f_0/f_s) + \mathrm{A}_s\mathrm{PRC}(\theta_i),$$

where PRC denotes the oscillator phase response curve and describes the change in the oscillator phase as a function of the stimulation phase. For the sine circle map, the PRC is given by $\mathrm{PRC}(\theta) = \sin(\theta)$.

By simulating the sine circle map, we are able to observe a 1:2 Arnold tongue (Fig S.4), which predicts 1:2 entrainment for a 75Hz oscillator at 130Hz stimulation. There exists a specific range of stimulation amplitudes for which we would expect to see 1:2 entrainment of the oscillator at a resultant frequency of 65Hz. This is in agreement with experimental observations [Swann et al., J. Neurosci., 2016] and provides theoretical grounds for expecting 1:2 entrainment during high-frequency stimulation.

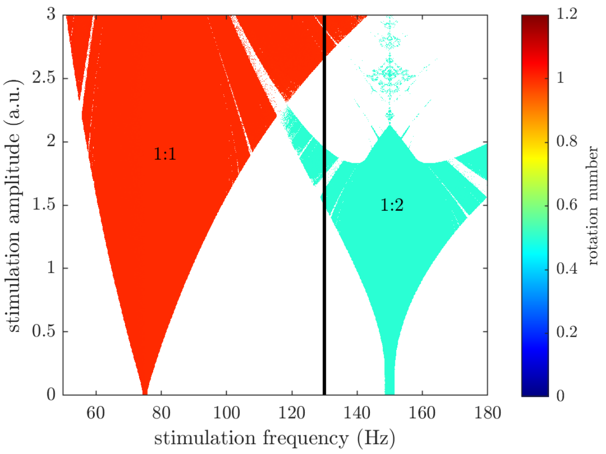


**Figure S.4: The sine circle map entrainment field.** Variable stimulation frequency with a fixed natural frequency of 75Hz. Only showing the 1:1 and 1:2 tongues for clarity, other higher order tongues are hidden.

While the sine circle map can provide a first-level description of gamma entrainment, its simplicity results in significant limitations. Firstly, as the oscillator stays on the unit circle, there is no variable amplitude of oscillations. This makes anything more than analysis of a single neuronal unit unreliable. Secondly, the sine circle map only represents a single oscillator. Therefore it is difficult to draw comparisons to ECoG signals that arise from interacting populations of neurons. Thirdly, it is known that pulse shape impacts entrainment behaviour; however, as the sine circle map is stroboscopic, realistic pulses cannot be used. Hence, a model which captures the interaction of neurons, is representative of larger neuronal populations and for which realistic pulse shapes can be used would be more suitable. We therefore consider an interacting neural populations model that is fitted to patient data to predict stimulation parameters that lead to 1:2 entrainment (See the *Wilson-Cowan Model* section in the main text).

# E Fitting Robustness

## E.1 Consistency Across Best Parameter Fits

The left lean of the tongue over the normalised stimulation amplitude axis is a consistent feature across the top model parameter fits. The example of RCS10 is shown in Fig S.5.

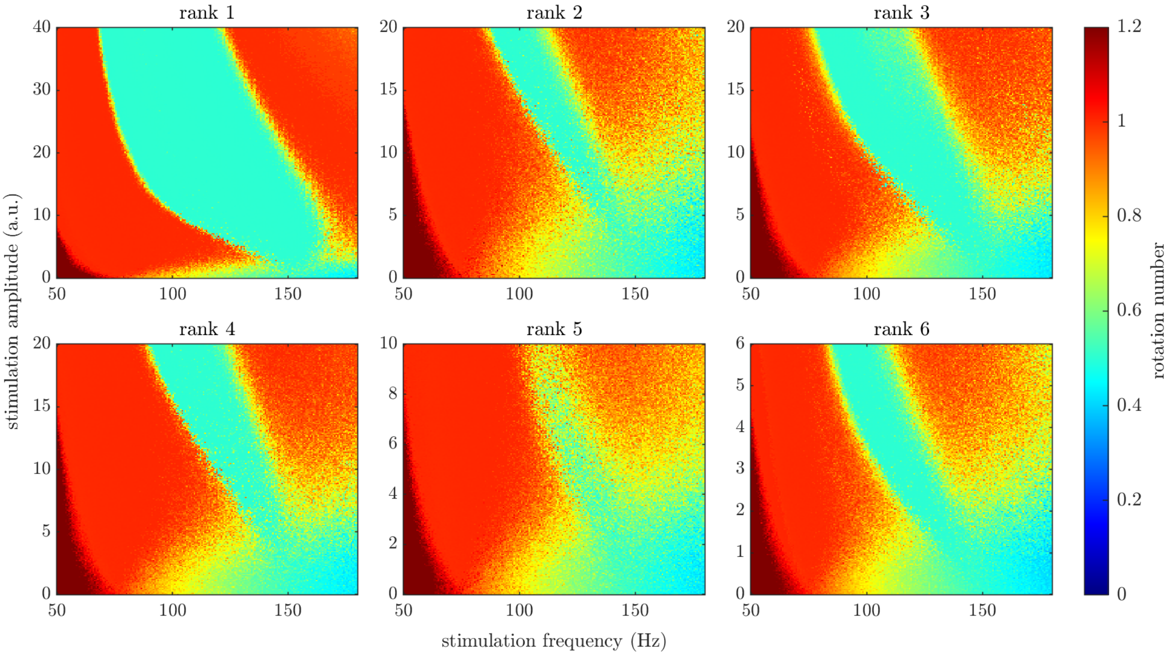


**Figure S.5: The entrainment fields for the top six ranked parameter fits of the Wilson-Cowan model to RCS10.** All model parameter fits presented here had an $R^2$ value greater than 0.92. Rank five's entrainment field is observed over a smaller range of stimulation parameters due to the smaller and noisy 1:2 tongue.

## E.2 Alternate Stimulation Pulse Shapes with Recharge All Predict Left Leaning 1:2 Tongues

Throughout this study, we have used the simplest pulse shape with no recharge. However, to achieve charge balancing at the site of stimulation, DBS requires a recharge component following the initial pulse. This component can be active when recharge is a set proportion of the stimulation period, or passive when charge is allowed to flow from the stimulation site via the electrode. The amplitude of the charge balancing component is calculated depending on the frequency of the stimulation and the amplitude of the positive pulse, so that the positive pulse and the negative charge balance have equal areas.

Across pulses with various recharge duration, the 1:2 tongue is present and exhibits a left lean as shown in Fig S.6 (example of RCS10). Despite these similarities, the tongues are present across different amplitude ranges. When recharge is present, the shortest recharge lengths require the greatest amplitude to observe the same extent of the 1:2 tongue. This is attributed to the immediate recharge cancelling the effect of the preceding pulse in the model.

Neurons are likely to respond to hyperpolarising and depolarising stimulation in different capacities. Immediately after a depolarising stimulation pulse, it is likely that a high proportion of the affected neurons will fire. The following hyperpolarising recharge will simply reduce membrane potential during the refractory period. Because of this asymmetrical effect and for simplicity, we chose to use a stimulation pulse with no recharge in this study. However, the prediction of a left leaning 1:2 tongue holds for pulses with recharge of various durations as shown in Fig S.6.

If the model does approximately capture the response of neurons to stimulation pulses with recharge, we would expect that passive recharge or recharge over a longer window would achieve entrainment at lower stimulation amplitudes. Entrainment at lower stimulation amplitudes would potentially increase the efficiency of stimulation protocols that are designed to entrain brain oscillations and minimise the side effects experienced from high stimulation amplitudes.

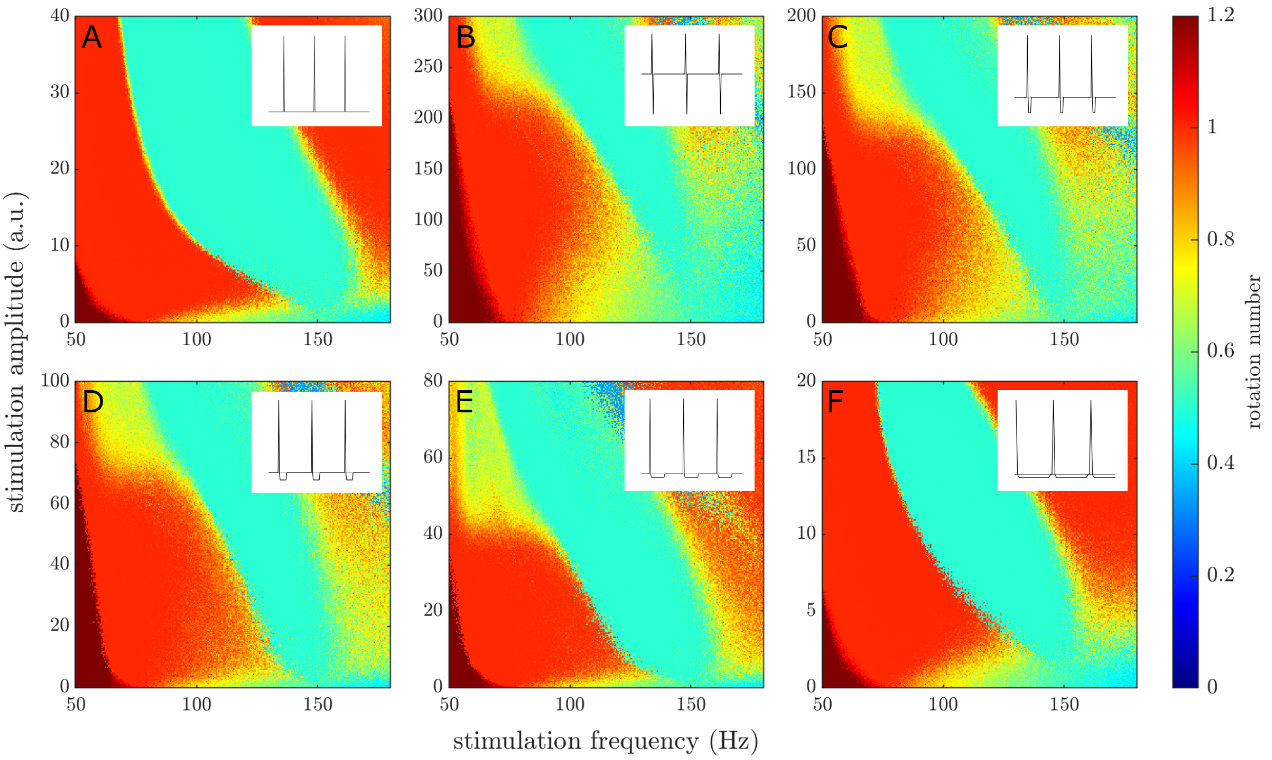


**Figure S.6: A comparison of six different stimulation pulse recharges applied to the inhibitory population of the top ranked Wilson-Cowan model fitted to RCS10.** The panels are categorised as a pulse with no recharge (A), which is the pulse used throughout the majority of the study, $100\mu s$ recharge (B), $400\mu s$ recharge (C), $1ms$ recharge (D), $2ms$ recharge (E), and whole period recharge (F), which can be used to approximate passive recharge. The inserts represent a couple of the periods of the corresponding stimulation pulse and the grey line indicates an amplitude of zero.

## E.3 Alternate Stimulation Waveforms Predict Different 1:2 Tongues

As the model is fitted to off-stimulation data, it is possible to apply a variety of stimulation waveforms to either population to predict entrainment responses. Fig S.7 shows the entrainment behaviour for RCS10 at high stimulation frequency for four waveforms: a single time step pulse with no recharge (the same as for Fig 6A, D, and G in the main text), a sine wave, a sawtooth wave, and a square wave. The amplitude scale for each waveform is normalised by the integral of the square of the waveform. This ensures that amplitude levels correspond to the same energy across the waveforms in Fig S.7. The sine wave and the single time step pulse have similar 1:2 tongues at frequencies greater than 150Hz, while the square and sawtooth waves are able to maintain 1:2 entrainment at higher stimulation amplitudes. Additionally, the three non-pulsatile waveforms are able to maintain 1:2 entrainment at lower stimulation frequencies and amplitudes than the pulsatile waveform. This is important to note as a patient may not be able to withstand the higher amplitudes on this scale without experiencing side effects.

## E.4 Tongue Lean and the Wilson-Cowan Vector Field

The left lean of the 1:2 tongue can be explained by analysing deterministic trajectories of the population activities in the E-I activity phase space. Fig S.8 shows that in the 1:2 entrainment region for RCS10, two positive pulses of stimulation occur for each circuit completion of the periodic trajectory (Fig S.8C). As stimulation amplitude and/or frequency are increased, the pulse that occurs at larger values of excitatory activity drops to lower values. Increased frequency (Fig S.8D) means less time for population dynamics to reach more extreme activities. Increased amplitude (Fig S.8A) pushes the activity further away from the fixed point and, hence, requires more time to reach the same value of excitatory population activity.

By considering stimulation parameters along the top right boundary of the 1:2 tongue, these effects

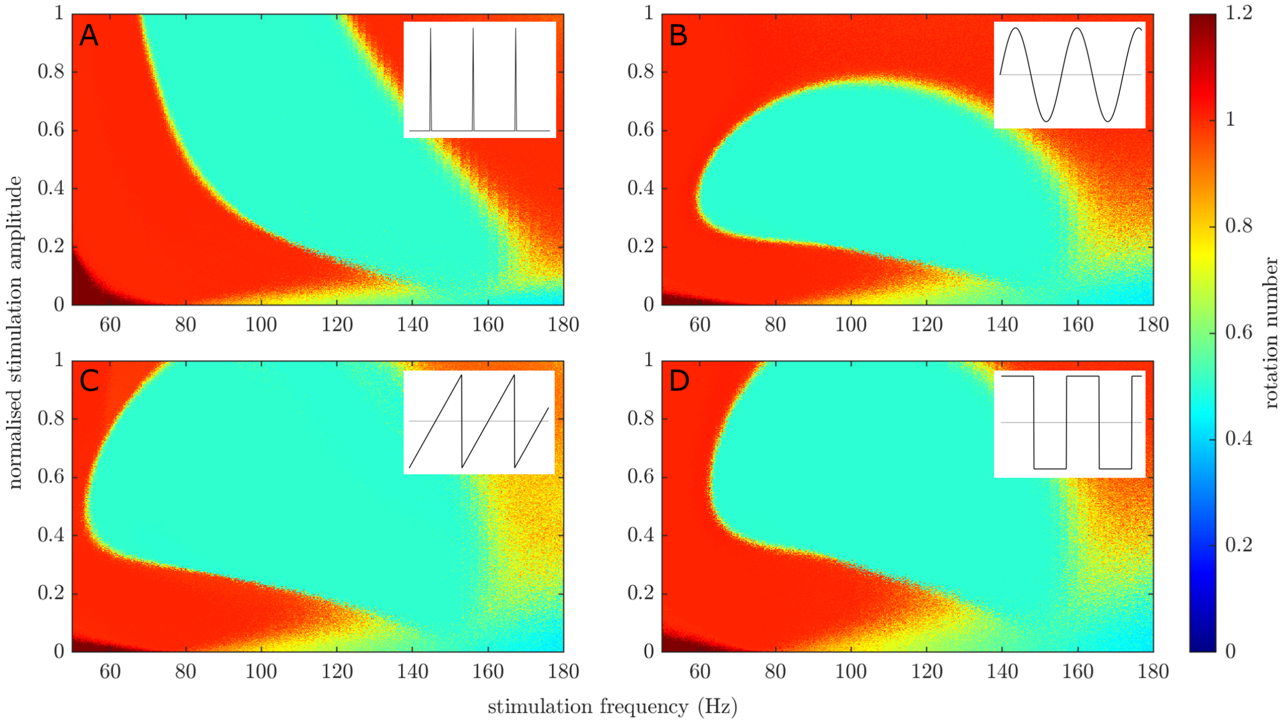


**Figure S.7: A comparison of four different stimulation waveforms applied to the top-ranked Wilson-Cowan model fitted to RCS10.** In each panel, stimulation takes the form of a single time step pulse with no recharge (A), a sine wave (B), a sawtooth wave (C), and a square wave (D). Stimulation amplitude is normalised against the integral of the square of the waveform. The inserts represent a couple of periods/cycles of the corresponding stimulation waveform and the grey line indicates an amplitude of zero.

can account for the left lean of the tongue. On the boundary, the pulse at higher excitatory population activity of the 1:2 entrained trajectory exactly crosses the fixed point in the absence of noise. In a noisy system it would cross either side of the fixed point, with these cycles corresponding to either 1:2 or 1:1 entrainment. From stimulation parameters on the boundary, changing one stimulation parameter requires mutually changing the other in the opposite direction to maintain this relationship of the higher excitatory activity pulse to the fixed point. For instance, if we increase stimulation frequency and there is less time between the successive positive pulses, we must decrease stimulation amplitude to maintain this relationship. This can be seen in the transition from trajectory A to D in Fig S.8. Hence, this gives us a relationship for the upper bound of the 1:2 tongue where the system transitions to 1:1 entrainment for increasing frequency or amplitude. As this is the upper bound of the tongue, this means that it must be left leaning.

The direction of the 1:2 tongue lean depends on the vector field. The entrained trajectory requires more time between pulses to reach the same location within the vector field as stimulation amplitude is increased. The rate of change of population activity is slowed down each period as it consistently reaches darker and slower regions of the colour-scaled angular phase velocity, as shown in the inserts of Fig S.8. If, however, the higher excitatory activity pulse reached a lighter coloured region of increased angular phase velocity, it would require less time between successive pulses to reach the same location within the vector field, i.e. increased stimulation frequency. Therefore, through differences in the vector field, for this hypothetical example, we would predict that increased amplitude would require an increase in frequency to maintain 1:2 entrainment. Unlike the results of our fitting process, this hypothetical example would produce a right leaning 1:2 tongue with a preference for speeding up the natural frequency.

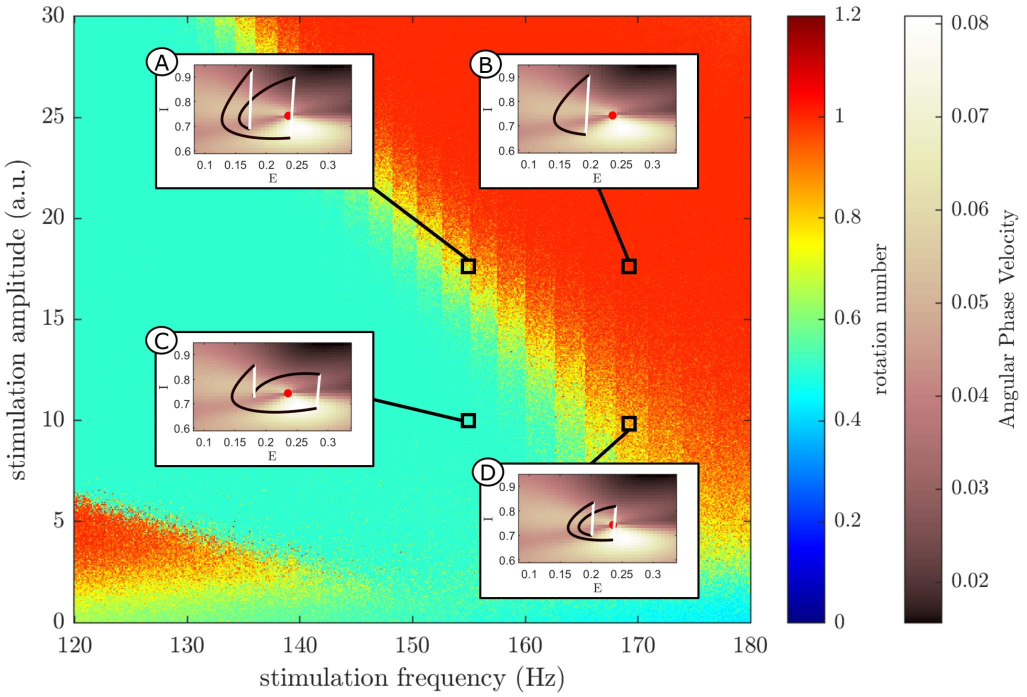


**Figure S.8: Deterministic trajectories in phase space and the left lean of the 1:2 tongue (example of RCS10).** On each of the four trajectory panels, the red dot represents the fixed point for the parameter set. The colourscale of the trajectory is chosen so that white represents a larger jump over an individual time step (following a stimulation pulse) and black is a smaller jump. The angular phase velocity colourbar indicates the magnitude of the vector field, with the colourscale of the entrainment field being identical to Fig 6B. Trajectory A (Amplitude = 18, Frequency = 155Hz) and D (Amplitude = 10, Frequency = 170Hz) show phase space trajectories of stimulation parameters on the 1:2 tongue boundary. Trajectory B (Amplitude = 18, Frequency = 170Hz) lies within the 1:1 tongue and trajectory C (Amplitude = 10, Frequency = 155Hz) is in the 1:2 tongue. The stimulation provided is a single time step pulse with no recharge over the rest of the period.

## E.5 Stimulating the Excitatory Population Instead of the Inhibitory Population

Throughout this paper, we stimulate the models consistently through the inhibitory population as described in the main text. However, the 1:2 tongue is still present when stimulating the excitatory population and while observing the inhibitory population activity (to avoid the population dynamics being dominated by stimulation artefact). Moreover, the key features of the 1:2 tongue are maintained regardless of the population being stimulated. As seen for RCS10 in Fig S.9 and Fig 6D, both 1:2 tongues originate at low amplitude from 150Hz stimulation and have a clear left lean towards lower frequencies. Conversely, stimulation of the excitatory population does not produce 1:2 entrainment at frequencies greater than 150Hz for a single time step pulse with no recharge. 1:2 entrainment above 150Hz was observed in both the model for stimulation of the inhibitory population, in Fig 6D, as well as in the data presented in Fig 6F. Furthermore, the 1:2 tongue resulting from stimulation of the excitatory population transitions to 1:1 entrainment at approximately one quarter the height of the 1:2 tongue of inhibitory stimulation. In the model, 1:2 tongues of similar characteristics originating from both the excitatory and inhibitory population is not a surprise. The network dynamics are responsible for the features of the 1:2 tongue. Therefore a strong periodic stimulus to either population will likely result in the network behaving with a similar response.

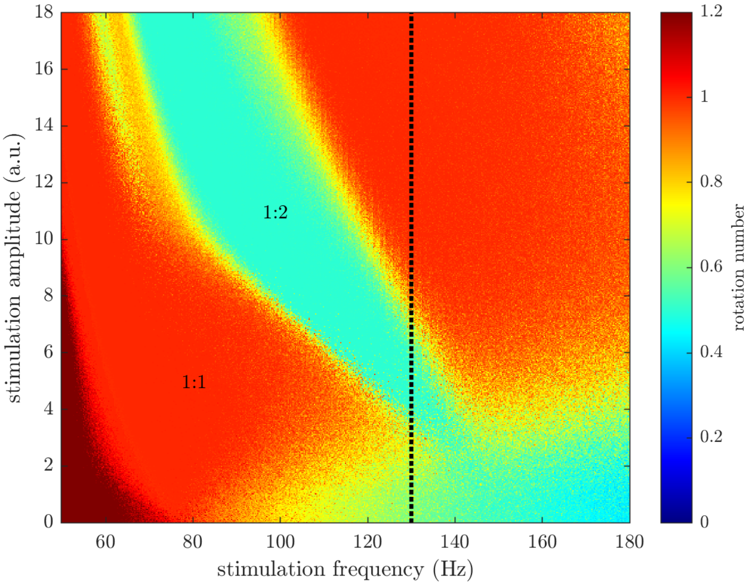


**Figure S.9: Entrainment field resulting from excitatory stimulation in the Wilson-Cowan model (top-rank fitted to RCS10).** Stimulation is for a single time step pulse with no recharge, but applied to the excitatory population and rotation number measured from the inhibitory population. The black dotted line is used to highlight the case of 130Hz stimulation frequency.

# F Entrained Signals Exhibit Phase-Locking

By the definition of entrainment, if oscillators are entrained to a signal then they must exhibit some level of phase-locking. This is observed differently for 1:1 and 1:2 entrainment. For pulsatile stimulation and a 1:1 entrained oscillator, every pulse will occur around the same phase as every cycle of stimulation is matched by a cycle of oscillations. For a 1:2 entrained oscillator, every oscillation cycle experiences two pulses of stimulation. This signal is still phase-locked, but every other pulse occurs around the same phase.

As different modes of entrainment are reflected in the phases at time of stimulation pulses, it is possible to categorise different entrainment regions by plotting these phases. While it is not possible to record from the same contact that is stimulating, it is possible to observe the stimulation artefact from the neighbouring subcortical contacts. By tracking these artefacts and verifying that they align with the stimulating frequency, it is possible to find approximate time stamps of the stimulating pulse

train. We record the corresponding phases by taking the Hilbert transform of the cortical signal at these time stamps. Prior to passing the signal through the Hilbert transform, the signals are band-passed between 70Hz and 80Hz for this 149.3Hz stimulation case. This isolates the potentially 1:2 entrained signal, the main observation of interest for this study. These results are plotted in Fig S.10.

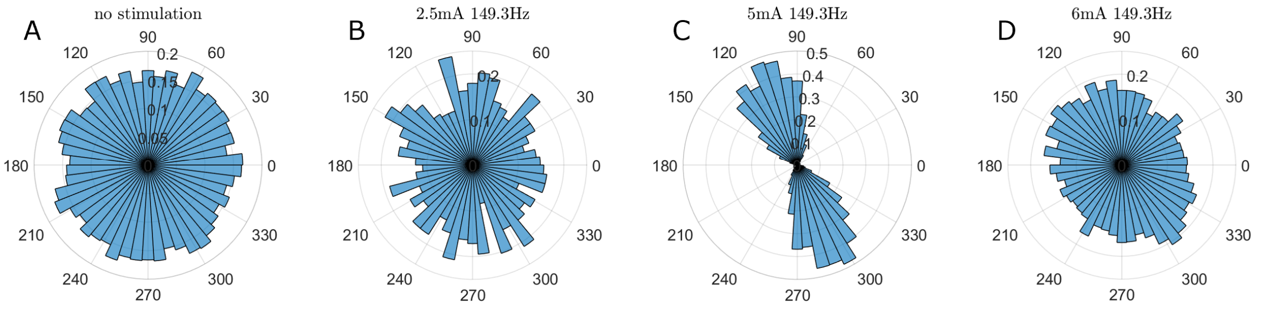


**Figure S.10: Cortical gamma phases at the time of stimulation for four different stimulation settings in RCS10.** Each panel is plotted as a polar histogram and normalised as a probability density function estimate where the sum of the area of the bars equals one. The signals were band-passed between 70 and 80Hz prior to identifying the phases. (A) No stimulation with sham stimulation triggers to record cortical gamma phase at 149.3Hz, (B) 2.5mA for the recording that did not show 1:2 entrainment, (C) 5mA where 1:2 entrainment was observed and (D) 6mA where 1:2 entrainment was not observed.

For Fig S.10A, off stimulation, the recording of phases was triggered by sham stimulation at 149.3Hz. As there is no stimulation, there is no entrainment in panel S.10A as shown by the absence of preferred phases. Panels S.10B and D did not display 1:2 entrainment in their respective PSDs and do not display preferred phases. In contrast, panel S.10C had a half harmonic peak above the predicted baseline in its PSD and was recorded as seeing 1:2 entrainment. Its corresponding polar histogram displays phases grouped around two, approximately polar opposite, phases. This is in agreement with the spectral evaluation and what we would expect to see from a 1:2 entrained signal.
